# Elevated Th1 and terminally differentiated cytotoxic T cells with suppressed Tc17 lymphocytes in lung tissue of advanced COPD and IPF patients undergoing lung transplantation

**DOI:** 10.3389/fimmu.2025.1646711

**Published:** 2025-10-14

**Authors:** Irena Šarc, Matija Rijavec, Luka Dejanović, Ana Koren, Matthias Zimmermann, Hendrik J. Ankersmit, Peter Korošec

**Affiliations:** ^1^ University Clinic of Respiratory and Allergic Diseases, Golnik, Slovenia; ^2^ Medical Faculty, University of Ljubljana, Ljubljana, Slovenia; ^3^ Biotechnical Faculty, University of Ljubljana, Ljubljana, Slovenia; ^4^ Christian Doppler Laboratory for Cardiac and Thoracic Diagnosis and Regeneration, Medical University of Vienna, Vienna, Austria; ^5^ Department of Oral and Maxillofacial Surgery, Medical University of Vienna, Vienna, Austria; ^6^ Division of Thoracic Surgery, Department of Surgery, Medical University of Vienna, Vienna, Austria; ^7^ Faculty of Pharmacy, University of Ljubljana, Ljubljana, Slovenia

**Keywords:** COPD, IPF, IPAH, Th1, Tc17, double-negative T cells, CD8^+^CD28^-^

## Abstract

**Introduction:**

The immunopathogenesis of end-stage chronic obstructive pulmonary disease (COPD) and idiopathic pulmonary fibrosis (IPF) remains poorly understood. Emerging evidence suggests that distinct T cell subpopulations may play critical roles in the progression of both diseases. A better understanding of these roles could provide important insights into underlying mechanisms and guide the development of targeted therapies.

**Methods:**

We performed flow cytometric analysis of explanted lung tissue from patients with advanced COPD (n = 9), IPF (n = 9), and idiopathic pulmonary arterial hypertension (IPAH, n = 3) undergoing lung transplantation. Healthy donor lung tissue (n = 7) served as controls.

**Results:**

Both COPD and IPF lungs demonstrated an increased frequency of Th1 (CXCR3^+^CCR4^-^CCR6^-^) lymphocytes compared to controls. In contrast, Tc17 cells were significantly reduced. No notable differences were observed in Th2, Th17, or Tc1 cell populations. Activated CD4^+^ T cells (CD69^+^CD25^+^HLA-DR^-/+^) were significantly enriched in IPF compared to COPD and donor lungs. COPD lungs exhibited a marked expansion of terminally differentiated cytotoxic CD8^+^CD28^-^CD27^^-^^ T cells. In double-negative (DN; CD3^+^CD4^-^CD8^-^) T cell compartment, CD25^+^ T cells were increased in COPD, whereas DN tissue-resident memory (TRM; CD69^+^CD25^-^HLA-DR^-^) cells were reduced in both COPD and IPF. Invariant natural killer T (iNKT; Vα24^+^Vβ11^+^) cell levels were uniformly low without intergroup differences.

**Discussion:**

Our findings identify disease-specific immune signatures in end-stage COPD and IPF. Th1 cell expansion together with a reduction in Tc17 and DN TRM subsets represented shared features of COPD and IPF, whereas accumulation of terminally differentiated cytotoxic CD8^+^ T cells and CD25^+^ DN T cells was specific to COPD. These findings enhance our understanding of adaptive immune dysregulation in COPD and IPF and may support the development of immunomodulatory strategies.

## Introduction

The pathogenesis of chronic obstructive pulmonary disease (COPD) and idiopathic pulmonary fibrosis (IPF) is not entirely understood (1). Although they are different conditions, COPD and IPF have several similarities. Both have a characteristic chronic and progressive course of lung destruction with a predominant incidence in the elderly male population. Both diseases are associated with exposure to long-term cigarette smoke and involve an abnormal response to repeated injury in the lung and dysregulation of the immune response. Currently, there is no cure for COPD and IPF (2).

In COPD, inhalation of cigarette smoke stimulates a chronic inflammatory response in the lungs, characterized by excessive accumulation of innate immune cells and activation of the adaptive immune system. Increased numbers of T lymphocytes in the lung tissue and airways of COPD patients have been shown at all stages of the disease, with a more significant increase in CD8^+^ lymphocytes than in CD4^+^ lymphocytes (2–5). The increased number of cytotoxic lymphocytes in COPD is proportional to the severity of the disease (3, 6). A greater proportion of CD8^+^ type 1 (Tc1) cells and greater expression of the cytotoxic effector proteins granzyme and perforin in CD8^+^ lymphocytes have been demonstrated in COPD patients (3, 7). Tc17 cells were enhanced in a mouse model of emphysema and associated with disease progression (8, 9). Some findings suggest immunological senescence in COPD patients, with an increased proportion of T lymphocytes lacking CD28 co-stimulatory receptor expression (CD4^+^CD28^-^ and CD8^+^CD28^-^ cells); these cells release higher amounts of perforins and granzymes and are more resistant to apoptosis (6) and potentially steroid-unresponsive (10). A previous study has shown high numbers of CD4^+^CD28null cells in lung tissue obtained from end-stage COPD patients, with lung-resident CD4^+^ T cells showing a proliferative response to extracellular matrix components (11). Additionally, in peripheral blood, elevated CD4^+^CD28null cells were found to induce increased cytokine production following stimulation of peripheral blood mononuclear cells (PBMCs) in early-stage patients with COPD (12). Mouse models of emphysema have shown significantly reduced alveolar destruction in the absence of cytotoxic T lymphocytes, suggesting their potential key role in the pathogenesis of emphysema (3). Furthermore, Th1 and Th17 cells have been shown to accumulate in the lungs of patients with stable COPD. An imbalance between proinflammatory and anti-inflammatory immune responses mediated by different subsets of CD4^+^ T lymphocytes, such as Th17 and regulatory Treg cells, has also been described in COPD patients, but their precise role is unclear (13, 14).

The role of inflammation in the development of IPF is a subject of ongoing research, and the involvement of lymphocytes in the onset and progression of IPF is still not understood. However, increased numbers of CD4^+^ and CD8^+^ T lymphocytes were found in the lung, and the number and proportion in the BAL of IPF patients were proportional to the severity of the disease (15). The pathogenesis of IPF has historically been attributed to an imbalanced Th1/Th2 immune response. Nevertheless, therapy against Th2 response has not been shown to be beneficial in IPF (15). The role of T lymphocytes in IPF is complex, as subpopulations of lymphocytes have a potentially profibrotic or antifibrotic effect. Most studies suggest that Th17 and CD8^+^ lymphocytes are likely to have a profibrotic effect, whereas CD4^+^ Th1 lymphocytes have a protective effect (15). Similar to COPD, increased numbers of CD28^-^negative CD8^+^ lymphocytes have been described in the lungs of IPF patients in a few studies (16). This subpopulation of lymphocytes has both a profibrotic and a proinflammatory transcriptional profile.

Lung transplantation is the only intervention shown to increase life expectancy and quality of life in patients with IPF (17) and end-stage COPD, and it is the final treatment option for selected patients (18). Our study aimed to analyze lung tissue lymphocyte subpopulations of COPD and IPF patients undergoing lung transplantation and compare them to those in the lung from pre-transplant donor lungs. Additionally, we included lung samples from a small group of individuals with advanced medically refractory idiopathic pulmonary artery hypertension (IPAH) undergoing lung transplantation. In these patients, the role of adaptive immunity, particularly T lymphocytes, remains largely unknown, with minimal data available.

## Materials and methods

### Study design and population

We prospectively enrolled patients with end-stage COPD, IPF, and IPAH undergoing lung transplantation, as well as a control group of lung donors. IPF was diagnosed by a multidisciplinary team following the 2011 guidelines of the American Thoracic Society and European Respiratory Society (19). COPD and IPAH diagnoses were made following current GOLD and IPAH guidelines at the time (20, 21). Patients were screened at transplant clinic visits, during which pulmonary function tests and clinical and laboratory data were obtained, and lung transplant eligibility was determined. Lung tissue samples from patients with advanced COPD, IPF, or IPAH were obtained from explanted lungs. A healthy lung sample was obtained from a portion of the pretransplant donor lung in cases where the donor lung had to be cut due to lung size mismatch. No patient or donor had an acute infection, and all relevant clinical data (lung disease, comorbidities, smoking status) were recorded for all the subjects prior to the procedure.

Patients were recruited at Allgemeines Krankenhaus (AKH) in Vienna, Austria, where lung tissue samples were collected at the Abteilung für Thoraxchirurgie (Department of Thoracic Surgery) during lung transplantation procedures performed between 9:00 AM and 5:00 PM. Samples were prepared in the Christian Doppler Laboratory for Cardiothoracic Diagnostics and Regeneration on the same day and then transported overnight to the Laboratory of Clinical Immunology and Molecular Genetics of the University Clinic of Pulmonary Diseases and Allergy Golnik, Slovenia, arriving at approximately 8:00 AM. Flow cytometry and cellular analysis were carried out on the same day.

The study protocol was approved by the Ethics Committee of the Medical University of Vienna (EK no. 091/2006) and the Slovenian National Medical Ethics Committee (KME 112/06/15). All procedures were conducted in accordance with the principles of the Declaration of Helsinki.

### Lung tissue sample preparation and flow cytometry

After sampling at AKH, the lung tissue samples, measuring approximately 6–8 mm × 6–8 mm and weighing 2 to 3.5 g were submerged in a transport medium consisting of ice-cold RPMI-1640 (Sigma–Aldrich, St. Louis, MO) supplemented with 1% bovine serum albumin (BSA, Miltenyi Biotec, Germany), 1% L-glutamine, and 1% penicillin-streptomycin (both from Sigma–Aldrich). The tissue was then finely minced into small pieces using a scalpel, washed, and enzymatically degraded with 20 µg/ml collagenase II (Gibco, USA) for 2 hours at 37°C with mixing every 15 minutes. The cell suspension was then filtered through a 70-μm nylon strainer (BD Falcon, USA), washed twice, resuspended in a transport medium, and shipped overnight on ice to the University Clinic of Pulmonary Diseases and Allergy Golnik, where flow cytometry analyses were performed the following day. The cells were first washed twice with PBS and stained for 15 minutes with CD4-fluorescein isothiocyanate (FITC) (clone SK3), CCR6-phycoerythrin (PE) (clone 11A9), CD3-PerCP (clone SK7), CCR4-PE/Cy7 (clone 1G1), CXCR3-allophycocyanin (APC) (clone 1C6), and CD8-APC/Cy7 (clone SK1) mAbs; with CD4-FITC, CD69-PE (clone L78), anti–HLA-DR-PerCP (clone L243), CD25-APC (clone 2A3) CD3-PE/Cy7 (clone SK7) and CD8-APC/Cy7 mAbs; with CD28-FITC (clone CD28.2), CD27-PE (clone M-T271), CD3-PerCP, and CD8-APC mAbs; and with Vβ11-FITC (clone C21), 6B11-PE (clone 6B11), CD3-PerCP, CD4-APC (clone SK3), and CD8-APC/Cy7 mAbs (all from BD Biosciences, San Diego, Calif). The stained cell samples were lysed, washed, and fixed (all the solutions were from BD Biosciences). Samples were acquired within 2 hours by a Canto II flow cytometer. 100,000 events were acquired in forward and side-scatter lymphocyte gates in each tube analyzed.

### Flow cytometry gating strategy and data analysis

**Figure 1:**
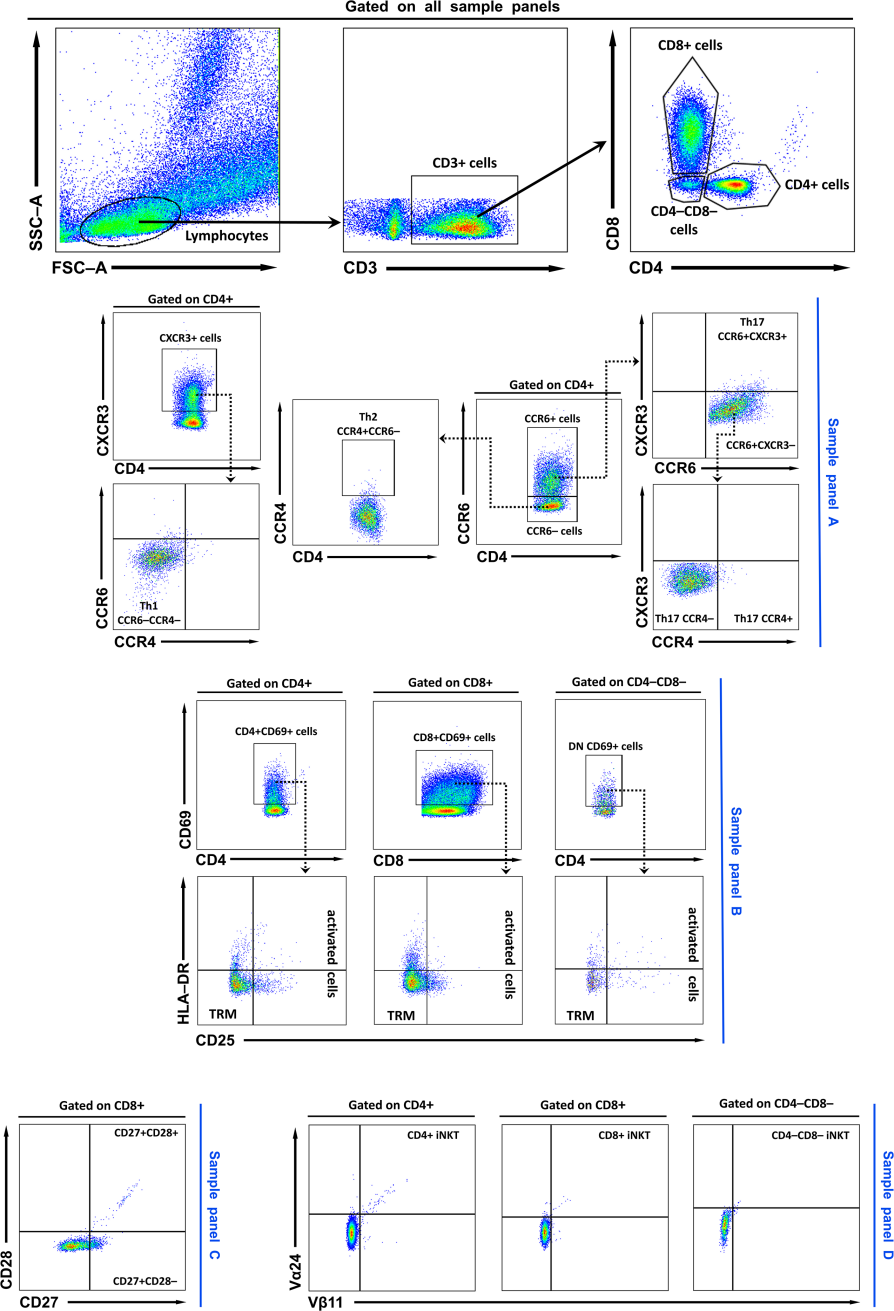
Representative FACS plots and gating strategy. Lymphocytes were first identified based on FSC/SSC profiles. Within the lymphocyte gate, T cells were defined as CD3^+^, and subsequently subdivided into CD4^+^, CD8^+^, or double-negative (CD4^–^CD8^–^; DN) subsets. In sample **(A)**, CD4^+^ and CD8^+^ cells were further characterised using CXCR3, CCR4, and CCR6 markers. Th1 cells were defined as CD4^+^CXCR3^+^CCR4^–^CCR6^–^, Th2 cells as CD4^+^CCR4^+^CCR6^–^ cells, and Th17 cells as CD4^+^CCR6^+^. Th17 cells were further subdivided into CXCR3^+^, CXCR3^–^CCR4^-^ and CXCR3^-^CCR4^+^ populations. In sample **(B)**, T cells were analysed for expression of activation and residency markers (CD69, HLA-DR, and CD25). TRM cells were gated as CD69^+^CD25^–^HLA-DR^–^, while activated T cells were defined as CD69^+^CD25^+^HLA-DR^+/–^. In sample **(C)**, T cells were evaluated for CD27 and CD28 expression. Finally, iNKT cells were identified in sample panel **(D)** by co-expression of Vβ11 and Vα24. TRM, tissue-resident memory T cells; DN, double negative (CD4^–^CD8^–^) T cells; Th, T helper cell; iNKT Invariant natural killer T cells.

Lymphocytes were identified on FSC/SSC plots, gated as CD3^+^, and subdivided into CD4^+^, CD8^+^, or double-negative (CD4^–^CD8^–^; DN) subsets. Subsets were analyzed using four staining panels: Panel A (CXCR3, CCR4, CCR6 for chemokine receptors), Panel B (CD69, HLA-DR, CD25 for activation and residency markers), Panel C (CD27 and CD28 for differentiation), and Panel D (Vβ11 and Vα24 for invariant NKT cells). The representative gating strategy is shown in **Figure 1**, with additional details provided in the figure legend. Cell viability data are provided in the **Supplementary Material** (**Supplementary Figure S1**). Flow cytometry data were analysed with BD FACSDiva software v6.1.3 (BD Biosciences) and FlowJo software v10 (BD Biosciences).

### Statistical analysis

Data are presented as the median and range or mean ± standard deviation (SD). The distribution of the data was assessed using the D’Agostino-Pearson omnibus normality test. For group comparisons involving COPD, IPF and healthy donor lung data, we used one-way analysis of variance (ANOVA) with Tukey’s *post-hoc* test for parametric data and the Kruskal–Wallis test, followed by Dunn’s *post hoc*-test for nonparametric data. Differences were considered significant at p < 0.05. Statistical analyses were performed using GraphPad Prism 9.4 (GraphPad Software, San Diego, California, USA).

## Results

### Patient and sample information

Explanted lung tissue samples from 9 patients with advanced COPD, 9 patients with advanced IPF, and 3 patients with IPAH were included in the analysis; for the control group, we included lung tissue samples from 7 lung donors (**Table 1**). COPD and IPF patients and donors did not differ significantly in age, with a mean age of 55 in both groups, compared with 48.6 (± 6.5) years in lung donors (p 0.16); patients with IPAH were younger (mean 39 years ± 13). Approximately half of the patients in the COPD and IPF groups were male; however, most lung donors (6 out of 7) were male, and all individuals with IPAH were female. The great majority of patients with COPD (8 of 9) and one-third of those with IPF (3 of 9) were ex-smokers; all lung donors and IPAH individuals were never smokers. Pulmonary function decline was more pronounced in COPD patients, with the majority (8 out of 9) receiving inhaled corticosteroid treatment at the time of inclusion (**Table 1**).

**Table 1:** Baseline characteristics of patients across groups.

Characteristics	COPD	IPF	IPAH	CONTROLS	P value*
Subject, No.	9	9	3	7	
Gender % (male)	55.6	55.6	0	86	0.37
Age, y	55.2 ± 8.5	55.1 ± 6.2	39.3 ± 13.0	48.6 ± 6.5	0.16
Smoking status: Ex-smoker Never smoker	88.9% 11.1%	37.5% 62.5%	66% 33%	0% 100	**0.001** **0.001**
Smoking preceding 6 months (%)	0	0	0	0	
FEV1, ml FEV1,% predicted	591 ± 239 19.2 ± 8.8	1505 ± 664 47.3 ± 15.6	2090 ± 823 57.0 ± 20.2	N/A N/A	**0.005** **0.003**
FEV1/FVC, %	31.5 ± 9.2	90.3 ± 11.8	81.0 ± 39.2	N/A	**0.001**
COPD Stage D n (%) Alpha-1 antitrypsin deficiency, n (%)	9 (100%) 1 (11.1%)	N/A	N/A	N/A	
Years since diagnosis	11.6 ± 13.2	4.6 ± 3.5	8.0 ± 0.0	N/A	0.102
BMI, kg/m^2^	23.1 ± 2.9	22.5 ± 5.5	23.3 ± 3.8	25.4 ± 0.9	0.19
Corticosteroid treatment (systemic/ICS) at the time of procedure	88.9%	12.5%	33%	100%	**>0.001**

COPD, Chronic obstructive pulmonary disease; IPF, idiopathic pulmonary fibrosis, IPAH, idiopathic pulmonary artery hypertension; FVC, Forced vital capacity; FEV1, Forced expiratory volume in first second; BMI, body mass index; ICS inhaled corticosteroids.

Data presented as mean ± SD, unlss indicated otherwise, comparisons with P value < 0.05 are in boldface.

### T cells, T helper cells, and cytotoxic T cells in lung tissue are increased in COPD and IPF

**Figure 2:**
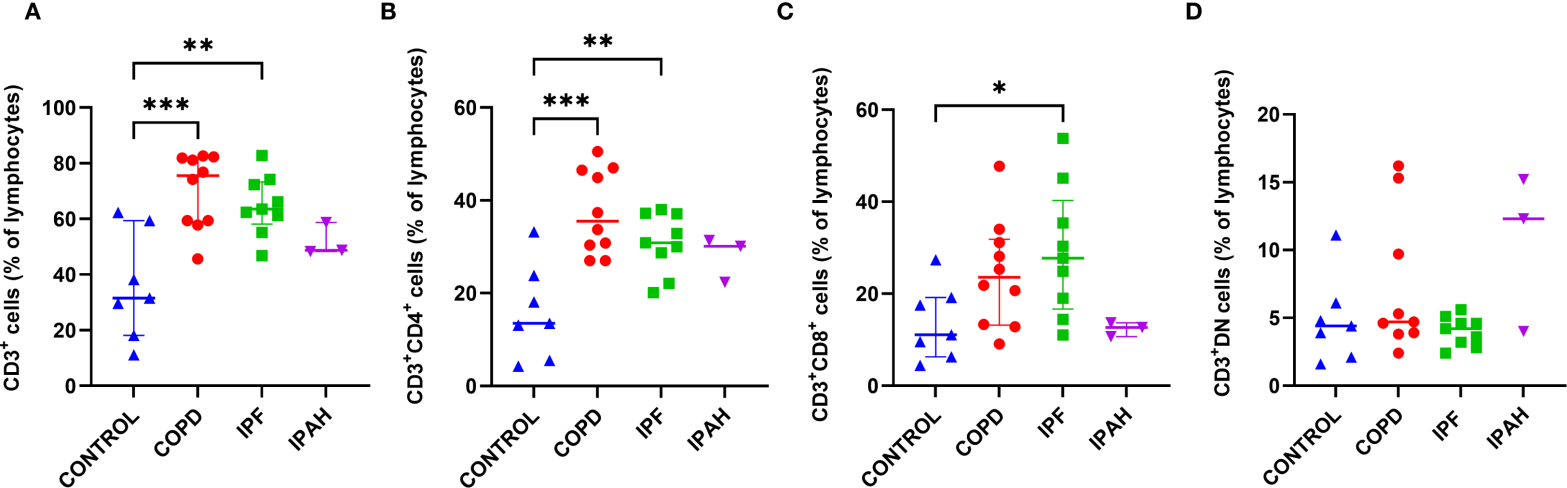
Subsets of CD3^+^ T cells in lung tissue across different patient groups, **(A-D)**. **(A)** Proportion of CD3^+^ T cells among total lymphocytes. **(B)** Proportion of CD3^+^CD4^+^ helper T cells. **(C)** Proportion of CD3^+^CD8^+^ cytotoxic T cells. **(D)** Proportion of CD3^+^ double-negative (DN) T cells (CD4^-^CD8^-^). p < 0.05 (*), p < 0.01 (**), and p < 0.001 (***).

To assess major subsets of lung lymphocytes, we first analyzed tissue T cells, T helper cells, and cytotoxic T cells. We found a strong predominance of CD3^+^ cells among lymphocytes in the lung tissue of COPD and IPF patients, with significantly higher values compared to healthy lungs of donors (**Table 2**, **Figure 2A**) (p≤ 0.0015; median [range]: 75.5% [45.6–82.3], 63.5% [46.8–82.8] *vs*. 31.5% [11.1–62.3], respectively). We then compared the percentages of CD4^+^ T cells and CD8^+^ T cells in the lung tissue of COPD and IPF patients with those in the donor’s lungs to determine the distributions of T cell subsets in COPD and IPF patients. There was a markedly 2.5-fold greater proportion of CD4^+^ cells in COPD and IPF lung tissues than in healthy lung tissue from donors (p ≤ 0.006; median [range]: 33.5% [27.0–50.5], 30.9 [20.1–38.0] and 13.5% [4.3–33.7], respectively) (**Table 2**, **Figure 2B**). Furthermore, the proportion of CD8^+^ T cells was significantly 2-fold greater in the lung tissue of IPF patients than in the healthy lung tissue of donors (**Table 2**, **Figure 2C**) (p= 0.04; 27.7% [11.0–53.8] *vs*. 11.1% [4.4–27.4]; however these differences did not reach significance in comparison with those in COPD patients (p= 0.17; 23.5% [9.1–47.7]). In a few individuals with IPAH, the T cell percentages more closely resembled those of healthy lung tissue donors than those of COPD or IPF patients (**Table 2**).

**Table 2:** T-cell surface parameters across diseases.

Tissue T-cell surface parameters	Healthy donor lung (n=7)	Disease groups of the explanted lung			Between groups	Group comparison		
		COPD (n=9)	IPF (n=9)	IPAH (n=3)	P-value	COPD *vs*. healthy	IPF *vs*. healthy	COPD *vs*. IPF
CD3^+^ cells (% of lymphocytes)	31.5 (11.1-62.3)	75.5 (45.6-82.3)	63.5 (46.8-82.8)	48.7 (48.3-58.7)	**0.0002**	**0.0002**	**0.0015**	0.72
CD3^+^CD4^+^	13.5 (4.3-33.7)	33.5 (27.0-50.5)	30.9 (20.1-38.0)	30.1 (22.4-31.4)	**0.0001**	**<0.0001**	**0.006**	0.22
CD3^+^CD8^+^	11.1 (4.4-27.4)	23.5 (9.1-47.7)	27.7 (11.0-53.8)	12.7 (10.7-13.7)	**0.047**	0.17	**0.04**	0.66
CD3^+^DN CD4- CD8^-^	4.4 (1.6-11.1)	4.7 (2.4-16.2)	4.2 (2.4-5.6)	12.3 (4.0 -15.2)	0.15	0.38	0.88	0.14
% of CD4^+^ cells								
CXCR3^+^	18.6 (0.7-28.7)	34.5 (12.4-49.8)	32.9 (23.5-50.2)	31.2 (12.9-51.9)	**0.033**	**0.039**	**0.003**	0.63
Th1 CXCR3^+^CCR4^-^ CCR6^-^	11.6 (7.48–27.0)	33.2 (12.4–48.3)	31.9 (24.0-50.5)	26.8 (13.2-51.1)	**0.0013**	**0.0048**	**0.0018**	0.9
CCR4^+^	2.6 (0.7-18.9)	2.2 (0.4-28.6)	1.6 (0.8-2.6)	1.2 (0.6-2.4)	0.61	0.75	0.43	0.48
Th2 CCR4^+^ CCR6^-^	1.4 (0.9–2.1)	2.0 (0.3–25.7)	1.3 (0.6–2.3)	1.0 (0.6–2.7)	0.8	>0.99	>0.99	>0.99
CCR6^+^	34.7 (20.3–97.1)	30.0 (11.6–46.4)	28.0 (20.6–44.7)	28.7 (15.9–40.1)	0.5	>0.99	>0.99	0.6
Th17 CXCR3^+^	1.3 (0.5–5.2)	0.9 (0.2–2.9)	1.1 (0.4–2.2)	1.4 (0.9–1.5)	0.6	>0.99	>0.99	>0.99
Th17 CXCR3^-^ CCR4^+^	0.7 (0.2–1.3)	0.4 (0.0–1.1)	0.3 (0.0–0.4)	0.9 (0.3–1.6)	0.1	>0.99	0.1	0.7
Th17 CXCR3^-^ CCR4^-^	31.7 (17.0–81.6)	27.1 (10.5–42.0)	26.1 (20.6–42.4)	26.4 (13.6–35.8)	0.5	0.8	>0.99	>0.99
% of CD4^+^ cells								
CD4^+^CD69^+^	62.5 (51.5-69.2)	56.0 (38.9-74.5)	60.1 (37.7-67.7)	73.2 (13.8-77.1)	0.47	0.66	0.99	0.99
CD4^+^CD25^+^	20.3 (8.9-29.9)	14.3 (5.9-21.9)	19.4 (7.5-31.5)	17.5 (5.6-18.0)	0.17	0.38	0.89	0.16
CD4^+^HLA-DR^+^	10.5 (5.5-54.7)	19.8 (6.5-59.2)	21.3 (10.4-39.4)	24.1 (5.1-24.9)	0.39	0.52	0.24	0.99
Activated CD4^+^ CD69^+^CD25^+^HLA- DR^-/+^	2.7 (0.6–10.5)	7.6 (2.8–10.1)	11.8 (9.2–20.4)	4.2 (2.0–6.3)	**0.0006**	0.58	**0.0006**	**0.03**
TRM CD4^+^ CD69^+^CD25^-^HLA- DR^-^	51.6 (24.9–59.2)	40.3 (22.1–48.3)	35.4 (16.5-53.4)	48.5 (10.7-62.9)	0.06	0.21	0.056	0.72
% of CD8^+^ cells								
Tc1 CXCR3^+^	0.3 (0.1–13.1)	0.1 (0.0 – 2.7)	0.2 (0.0–3.9)	0.2 (0.0–0.6)	0.11	0.1	0.74	0.32
Tc17 CCR6^+^	49.8 (37.8-70.6)	6.2 (1.4-58.2)	9.5 (6.1-54.5)	15.1 (12.0–39.4)	**0.01**	**0.02**	**0.047**	0.45
% of CD8^+^ cells								
CD8^+^CD69^+^	36.9 (20.6-78.9)	62.7 (41.4-81.8)	50.6 (26.6-85.4)	46.2 (20.9-87.4)	0.23	0.22	0.40	0.9
CD8^+^CD25^+^	4.4 (0.6-23.1)	2.9 (0.2-4.8)	3.6 (0.5-10.7)	1.0 (0.8-1.2)	0.49	0.73	0.99	0.99
CD8^+^HLA-DR^+^	14.1 (6.7-37.6)	22.8 (4.7-50.7)	26.2 (9.2-54.3)	18.3 (6.5-36.5)	0.18	0.33	0.17	0.89
Activated CD8^+^ CD69^+^CD25^+^HLA- DR^-/+^	2.2 (0.0–10.4)	1.6 (0.0–3.7)	1.4 (0.2–6.0)	1.05 (0.5–1.2)	0.94	>0.99	>0.99	>0.99
TRM CD8^+^ CD69^+^CD25^-^HLA- DR^-^	28.1 (17.1–57.1)	28.7 (21.5–36.7)	26.7 (20.5–70.6)	19.0 (16.4–66.9)	0.98	>0.99	>0.99	>0.99
% of CD8^+^ cells								
CD8^+^CD28^+^CD27^+^	0.1 (0.0-0.8)	0.1 (0.0-1.0)	0.1 (0.0-1.3)	0.1 (0.0-0.4)	0.70	0.96	0.46	0.48
CD8^+^CD28-CD27^+^	12.5 (0.0-52.5)	0.5 (0.0-4.8)	1.3 (0.4-3.3)	0.2 (0.0-0.9)	**0.022**	**0.015**	0.19	0.21
CD8^+^CD28-CD27-	87.5 (47.3-99.9)	98,9 (65.1-100.0)	98.7 (82.4-99.9)	99.3 (99.1-99.9)	**0.036**	**0.036**	0.18	0.38
% of DN T cells								
DN CD4-CD8-CD69^+^	70.3 (38.8-83.8)	56.6 (36.9-63.1)	59.5 (27.9-76.0)	60.7 (30.6-66.0)	0.15	0.13	0.53	0.57
DN CD4-CD8-CD25^+^	3.0 (0.2-6.0)	9.4 (3.4-19.6)	2.7 (1.0-8.9)	9.2 (6.9-10.1)	**0.005**	**0.009**	0.91	**0.015**
DN CD4-CD8-HLA- DR^+^	7.0 (3.3-46.0)	14.6 (4.3-22.7)	20.3 (10.0-31.8)	17.9 (4.4-23.8)	0.44	0.98	0.59	0.45
Activated DN CD69^+^CD25^+^HLA- DR^-/+^	1.4 (0.0–3.5)	3.6 (0.7–8.0)	2.7 (1.3–6.8)	2.4 (2.3–3.9)	0.09	0.09	0.18	0.92
DN TRM CD69^+^CD25^-^HLA- DR^-^	66.8 (34.5–77.5)	45.0 (29.8–51.7)	45.5 (34.7–55.7)	49.3 (24.2–61.8)	**0.02**	**0.04**	**0.04**	1.0
iNKT CD3^+^Vα24^+^ Vβ11^+^	0.6 (0.2-16.5)	0.8 (0.2-3.1)	0.6 (0.1-2.3)	1.0 (0.6-3.9)	0.69	0.87	0.63	0.45
iNKT CD3^+^CD4^+^ Vα24^+^ Vβ11^+^	0.1 (0.0-0.5)	0.1 (0.0-0.4)	0.1 (0.0-1.4)	0.3 (0.0-0.5)	0.85	0.87	0.78	0.81
iNKT CD3^+^CD8^+^ Vα24^+^ Vβ11^+^	0.1 (0.0-0.8)	0.1 (0.0-2.2)	0.0 (0.0-1.6)	0.0 (0.0-0.3)	0.87	0.91	0.69	0.46
iNKT CD3^+^CD4^-^CD8^-^ Vα24^+^ Vβ11^+^	0.4 (0.2–16.4)	0.8 (0.2–2.9)	0.5 (0.0–1.2)	0.8 (0.5–3.4)	0.4	>0.99	>0.99	0.6

COPD, Chronic obstructive pulmonary disease; IPF, idiopathic pulmonary fibrosis, IPAH, idiopathic pulmonary artery hypertension; TRM, tissue-resident memory T cells; DN, double negative (CD4^–^CD8^–^) T cells; Th, T helper cell; iNKT Invariant natural killer T cells.

Values are expressed as medians (range); P values < 0.05 are in boldface, IPAH patients not included into group comparison.

### Lung tissue Th1 lymphocytes were predominant in both COPD and IPF and were markedly higher than in donors

**Figure 3:**
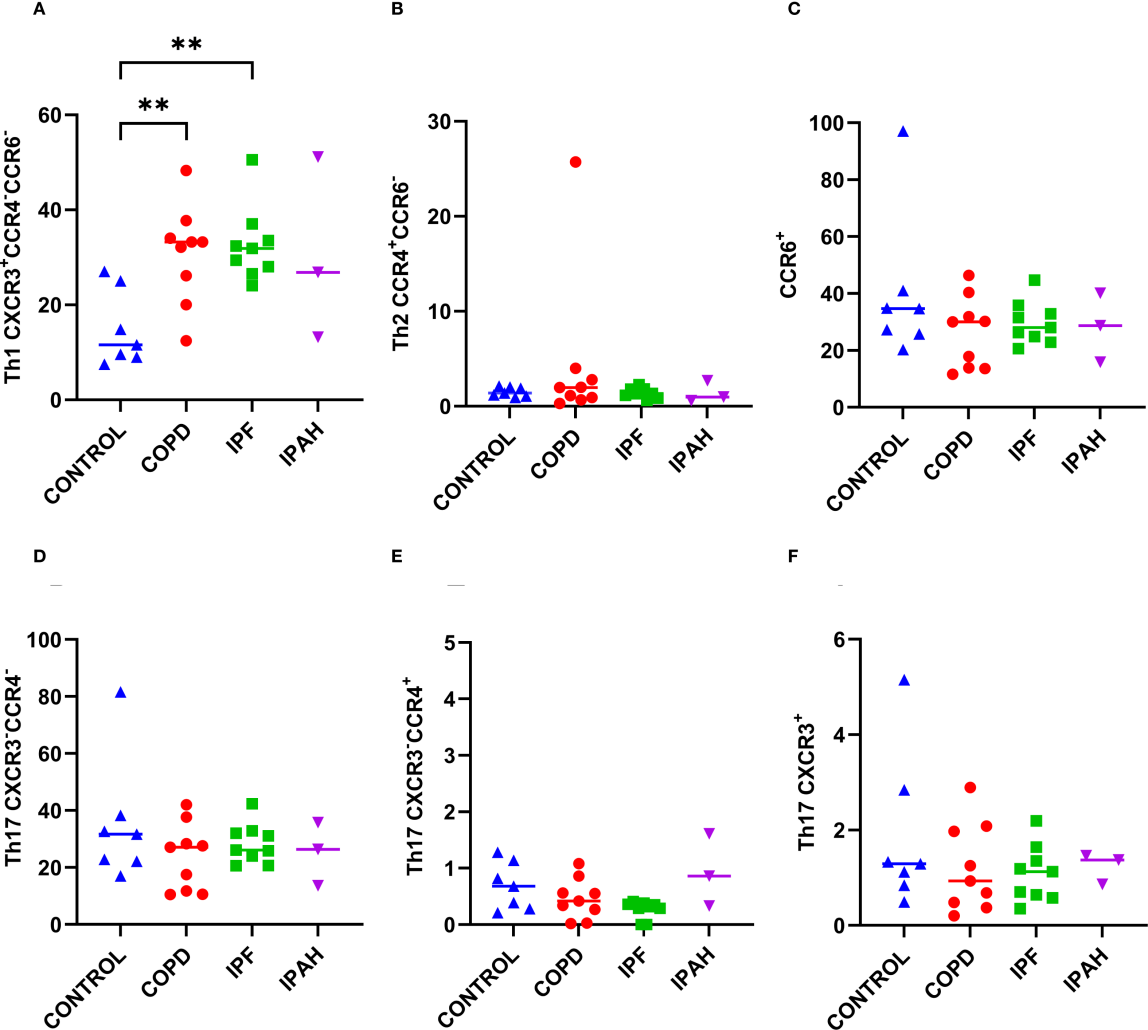
Subsets of CD4^+^ T cells in lung tissue across different patient groups, A-E. **(A)** Proportion of Th1 CXCR3^+^CCR4^-^CCR6^-^ cells among CD4^+^ T cells. **(B)** Proportion of Th2 CCR4^+^CCR6^-^ cells among CD4^+^ T cells. **(C)** Proportion of CCR6^+^cells among CD4^+^ T cells. **(D)** Proportion of Th17 CXCR3^-^CCR4^-^ cells among CD4^+^ T cells. **(E)** Proportion of Th17 CXCR3^-^CCR4^+^ cells among CD4^+^ T cells. **(F)** Proportion of Th17 CXCR3^+^. *p* < 0.05 (*), *p* < 0.01 (**).

To determine the proportions of Th1, Th2, and Th17 in a subset of CD4^+^ cells in lung tissue, we performed flow cytometric measurements of the surface expression of the chemokine receptors CXCR3, CCR4, and CCR6 (**Table 2**, **Figures 3A-E**). Both COPD and IPF patients exhibited approximately 2.9-fold higher percentage of CD4^+^ lung tissue Th1 CXCR3^+^CCR4^-^CCR6^-^ cells in comparison to Th1 tissue cells in healthy donor lungs (**Table 2**, **Figure 3A**) (p ≤ 0.0048; 33.2% [12.4–48.3], 31.9% [24.0–50.5] *vs*. 11.6% [7.48–27.0]), respectively). Additionally, the proportion of total CD4^+^ T cells in lung tissue was approximately 2.5-fold higher in COPD and IPF patients compared to donors (**Table 2**, **Figure 2B**); those differences demonstrate that overall, lung tissue of patients with COPD and IPF had approximately 7.2-fold higher fraction of Th1 lymphocytes than donors and thus highly induced type 1 immunity. In contrast, the proportions of CD4^+^ lung tissue Th2 CCR4^+^CCR6^-^ cells were comparable between groups and ranged between 1.3–2.0% [0.3–25.7]. In addition, we assessed the proportions of CCR6^+^ CD4^+^ T cells, which represented the most abundant population within the CD4^+^ compartment. Their proportions in lung tissue were comparable across groups, ranging from 28.0% to 34.7% [11.6–97.1]. Further subdivision of the CCR6^+^ compartment into phenotypically distinct Th17 subsets again revealed no significant differences between COPD patients, IPF patients, and healthy donors (**Table 2**, **Figures 3D, E**). Th17 (CXCR3^+^) cells ranged from 0.9% to 1.3% [0.2–5.2], Th17 (CXCR3^-^CCR4^+^) cells from 0.3% to 0.7% [0.0–1.3], and Th17 (CXCR3^-^CCR4^-^) cells from 26.1% to 31.7% [10.5–81.6].” Similar to COPD and IPF, IPAH patients demonstrated increased lung tissue Th1 lymphocytes (**Table 2**).

### Activated CD4^+^ T cells were increased in IPF, whereas lung tissue T-cell residency was comparable between patients and donors

We examined the expression of immune activation markers (CD69, CD25, and HLA-DR) on lung tissue CD4^+^ and CD8^+^ T cells. CD69, an early activation marker and hallmark of tissue-resident memory (TRM) cells in the lung, was expressed on approximately two-thirds of lung tissue T lymphocytes. The percentages of CD4^+^CD69^+^ (56% to 62.5% [37.7–74.5]) and CD8^+^CD69^+^ (36.9–62.7% [20.6–85.4]) were highly comparable between COPD, IPF and healthy donor lungs (**Table 2**). Similarly, CD25 marks T-cell activation and is also expressed on regulatory T cells; no significant differences were observed for CD4^+^CD25^+^ (14.3% to 20.3% [5.9–31.5]) or CD8^+^CD25^+^ cells (2.9% to 4.4% [0.2–23.1]).

To further delineate the CD69^+^ T cell compartment, cells were stratified based on CD25 and HLA-DR expression. CD69^+^CD25^-^HLA-DR^-^ cells were classified as TRM, while CD69^+^CD25^+^ and/or HLA-DR^+^ cells were considered activated. TRM CD4^+^ (CD69^+^CD25^-^HLA-DR^-^) and TRM CD8^+^ (CD69^+^CD25^-^HLA-DR^-^) cells constituted the majority of CD69^+^ CD4^+^ and CD8^+^ T cells and were comparably represented across COPD, IPF, and healthy donor lungs (35.4–51.6% [16.5–59.2] and 26.7–28.7% [17.1–70.6], respectively; **Table 2**). However, activated CD4^+^ T cells (CD69^+^CD25^+^HLA-DR^-/+^) were significantly increased in IPF compared to COPD and donor lungs (p = 0.0006; 11.8% [9.2–20.4] *vs*. 7.6% [2.8–10.1] and 2.7% [0.6–10.5], respectively; **Table 2**). Activated CD8^+^ T cells (CD69^+^CD25^+^HLA-DR^-/+^) showed no significant differences among IPF, COPD, and donor lungs (**Table 2**). IPAH patients’ residency and activation values were comparable to those of other patient groups and donors, with activated CD4^+^ T cells (CD69^+^CD25^+^HLA-DR^-/+^) similar to those of the controls (**Table 2**).

### Terminally differentiated cytotoxic CD28^-^CD27^-^ lung tissue T lymphocytes were increased in COPD

**Figure 4:**
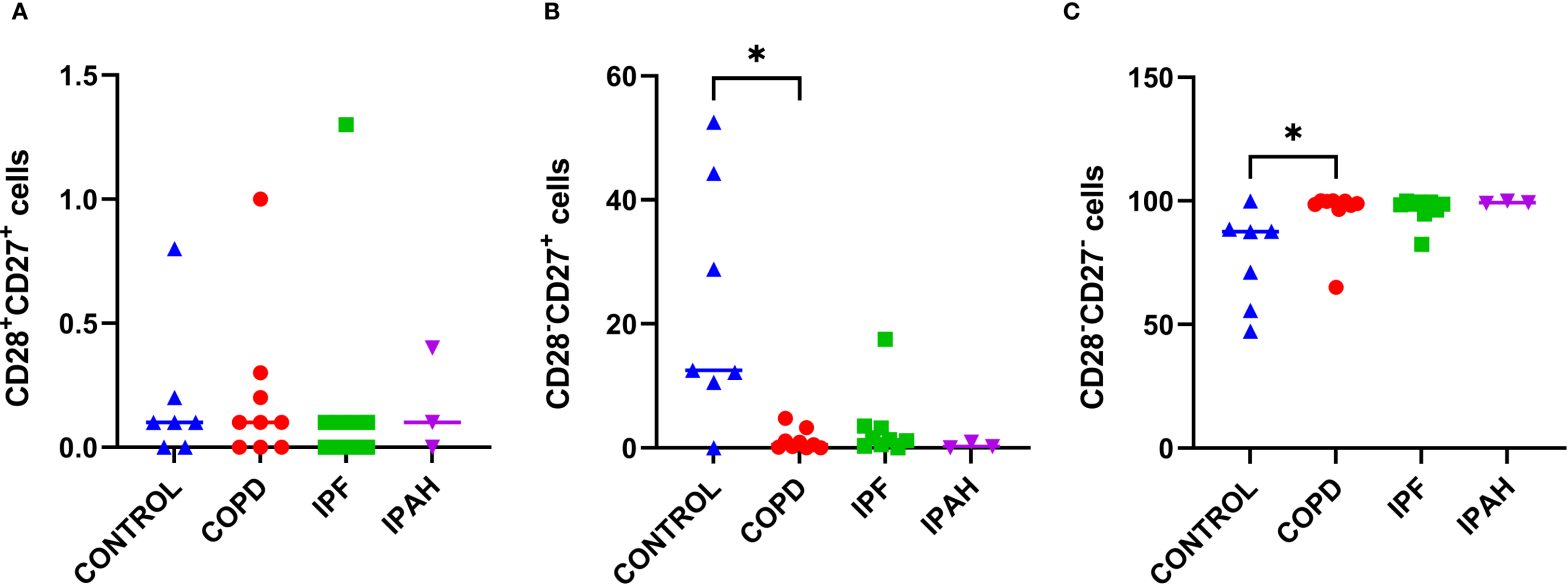
Subsets of cytotoxic CD8^+^ cells in lung tissue across different patient groups, **(A-C)**. **(A)** Proportion of CD28^+^CD27^+^ CD8^+^ T cells among CD8^+^ T cells. **(B)** Proportion of CD28^+^CD27^−^ CD8^+^ T cells among CD8^+^ T cells. **(C)** Proportion of CD28^−^CD27^−^ CD8^+^ T cells among CD8^+^ T cells. p < 0.05 (*).

We performed flow cytometric measurements of CD27 and CD28 surface molecule expression to study differences in the cytotoxic T-cell differentiation status (**Figure 4**). We found that the lung tissue of patients with COPD had a greater proportion of terminally differentiated CD8^+^CD28^-^CD27^-^ cytotoxic cells, than healthy lung tissue of donors (**Table 2**, **Figures 4B, C**) (p= 0.036; 98.9% [65.1–100] *vs*. 87.5% [47.3–99.9], respectively) and, consequently a lower proportion of partly differentiated CD8^+^CD28^-^CD27^+^ cells (P = 0.015: 0.5% [65.1–100] *vs*. 12.5% [0–52.5], respectively). Similar trends were observed in IPF patients (**Table 2**, **Figures 4B, C**), with 98.7% [82.4–99.9] being terminally and 1.3% [0.4–3.3] being partly differentiated; however, these differences did not reach statistical significance. Interestingly, IPAH patients also showed high terminally differentiated cytotoxic T-cell values (**Table 2**).

### Tc17 cells were highly reduced in the lung tissue of COPD and IPF

To further investigate the role of type 3 immunity in terminal COPD and IPF patients, we focused on T_C_17 cells and T_C_1 cells. Therefore, we determined the surface expression of the chemokine receptors CCR6 and CXCR3 on CD8^+^ T cells. We observed significant suppression of type 3 immunity, with more than 5-fold lower percentages of Tc17 cells in COPD and IPF lung tissue than in healthy lung tissue (P≤ 0.047; 6.2% [1.4–58.2], 9.5% [6.1–54.5] *vs*. 49.8% [0.7–28.7], respectively; **Table 2**). However, there was no difference for Tc1 cells, which were very low overall (0.1% to 0.3% [0.1–13.1]) among all groups.

### Lung tissue DN CD25^+^ T cells were increased in COPD, whereas DN TRM T cells were reduced in both COPD and IPF

**Figure 5:**
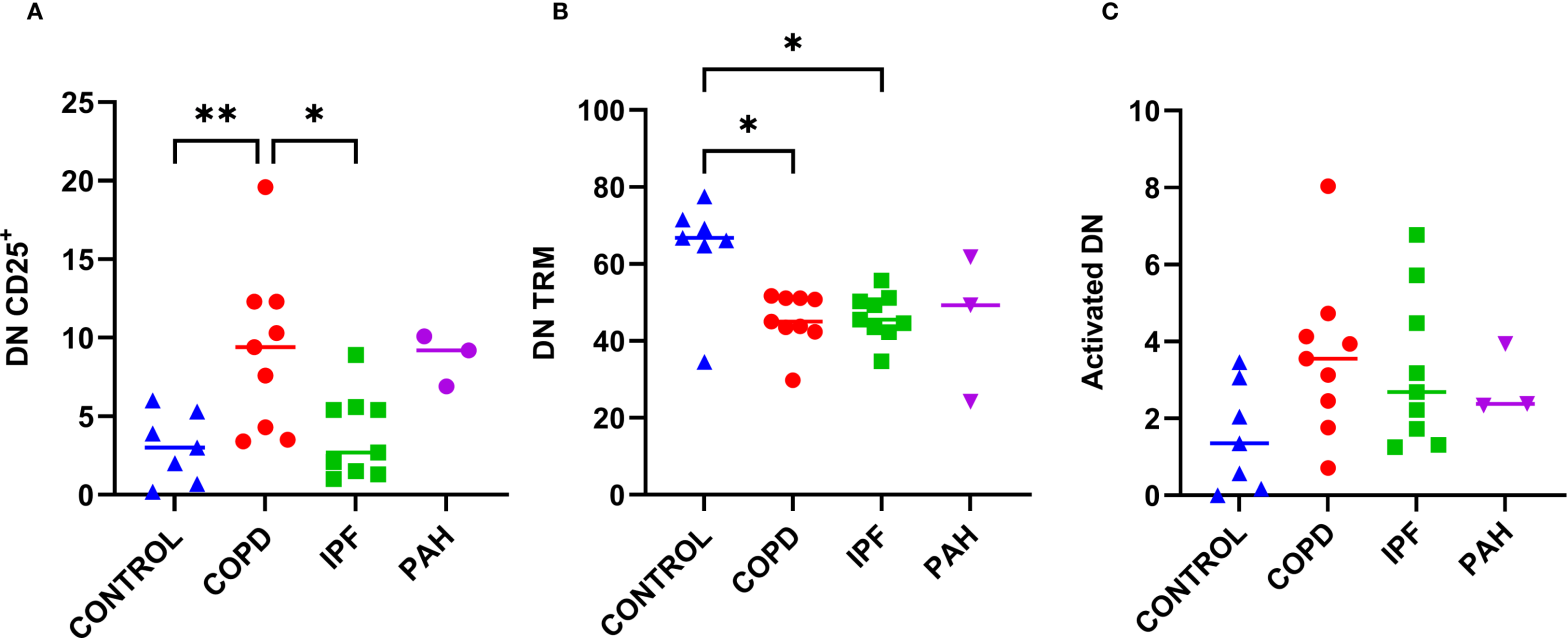
Proportions of CD25^+^, TRM, and activated double-negative T cells (CD3^+^CD4^–^CD8^–^; DN) among DN population in lung tissue across different patient groups, A–C. **(A)** Proportion of CD25^+^ DN cells. **(B)** Proportion of DN TRM cells (CD69^+^CD25^-^HLA-DR^-^). **(C)** Proportion of activated DN cells (CD69^+^CD25^+^HLA-DR^+/-^). p < 0.05 (*), p < 0.01 (**).

Previous reports have shown that DN T cells play a potential role in many inflammatory conditions (22). To explore the possible role of DN T cells in the pathogenesis of advanced IPF or COPD, we assessed their proportion within CD3^+^ cells, activation, and residency status in lung tissue. The overall proportion of DN T cells within CD3^+^ cells was comparable across COPD, IPF, and donor lungs (from 4.2% to 4.7% [1.6–16.2], **Table 2**, **Figure 1D**). In contrast, COPD patients exhibited higher proportions of DN CD25^+^ T cells than IPF patients or donors (p ≤ 0.015; 9.4% [3.4–19.6] *vs*. 2.7–3.0% [0.2–8.9]; **Table 2**, **Figure 5A**). DN CD69^+^ cells were the most abundant subset (56.6–70.3% [27.9–83.8]) and did not differ significantly between groups. As with CD4^+^ and CD8^+^ T cells, DN CD69^+^ cells were further subdivided into DN TRM (CD69^+^CD25^-^HLA-DR^-^) and activated DN (CD69^+^CD25^+^HLA-DR^-/+^) subsets. DN TRM cells were significantly more frequent in donor lungs compared to COPD and IPF (p < 0.04; 66.8% [34.5–77.5] *vs*. 45.0% [29.8–51.7] and 45.5% [34.7–55.7], respectively; **Table 2**, **Figure 5B**), whereas no significant differences were observed in activated DN T cells (**Table 2**, **Figure 5C**). IPAH individuals also exhibited higher frequencies of DN CD25^+^ T cells, similar to those observed in COPD.

### The level of lung tissue invariant NKT cells was low and not different between patients and donors

Some previous studies have suggested the importance of iNKT cells in lung diseases (23). However, in our patients with end-stage COPD and IPF, the levels of iNKT cells were low and highly comparable to those of healthy donor lungs (0.6% to 0.8% [0.1-16.5] for iNKT cells; 0.1% [0.0–1.4] for CD4^+^iNKT cells, 0% to 0.1% [0.0–2.2] for CD8^+^iNKT cells and 0.4 to 0.8% [0.0–16.4] for DN iNKT cells; **Table 2**).

## Discussion

In this study, we report several novel findings that advance the understanding of adaptive immune responses in end-stage IPF and COPD. Both diseases displayed a predominant Th1 signature in lung tissue at the pre-transplant stage, with no differences in Th2, Th17, or Tc1 subsets. COPD lungs were further distinguished by an accumulation of terminally differentiated cytotoxic CD8^+^ T cells and an increase in CD25^+^ DN T cells. In contrast, a reduction in Tc17 cells and DN TRM subsets was observed in both COPD and IPF, representing shared features of immune dysregulation.

We found compelling evidence that Th1 immunity links both severe COPD and IPF. This novel and unexpected finding, coupled with the observation of no differences in Th2 cells, may have important implications for understanding the pathogenesis of end-stage COPD and IPF, as well as for predicting the response to therapy. While Th1 cells have previously been shown to accumulate in the lungs of patients with stable COPD (24, 25), the finding in IPF was unexpected. The pathogenesis of IPF has historically been attributed to an imbalanced Th1/Th2 immune response, with Th2 being characterized as profibrotic and Th1 as protective (15). This paradigm has been challenged recently, as several interventional studies targeting the Th2 response in IPF patients were negative, with some agents having detrimental effects (15, 26). Additionally, in our IPF cohort, CCR6^+^CD4^+^ T cells predominated over CCR4^+^ cells, in contrast to the study of Adegunsoye et al. (27), who observed higher CCR4^+^ frequencies and CCR4:CCR6 ratio associated with less advanced disease. This discrepancy may reflect more advanced disease in our cohort or the inherent heterogeneity of the IPF population.

Our study is the first to demonstrate an increased frequency of terminally differentiated effector CD8^+^CD28^-^CD27^-^ cytotoxic T cells among CD8^+^ T lymphocytes in the lung tissue of patients with COPD. The loss of CD28 expression is a key indicator of premature lymphocyte senescence, typically driven by persistent immune activation (28). Unlike CD28^-^ cells in chronic viral infection, which often lack perforin and exhibit limited cytotoxicity, CD28^-^ cells in COPD display elevated levels of cytotoxic mediators, such as perforin and granzyme (29, 30), suggesting a potential role in nonspecific tissue damage. Studies have shown increased expression of cytotoxic effector proteins, including granzyme and perforin, in CD8^+^ lymphocytes in COPD lung tissue (3, 7). Similarly, several studies have reported an increase in CD4^+^CD28^-^ lymphocytes in the peripheral blood of COPD patients, along with elevated levels of intracellular perforin and granzyme B; however, findings are inconsistent (12, 31). One study observed an increase in CD4^+^CD28^-^ cells in end-stage COPD lung tissue, with purified lung-resident CD4^+^ cells exhibiting a stable proliferative response to lung-specific elastin and collagen (11). In our study, we focused on the CD8^+^CD28^-^ subpopulations, for which the increase in the COPD lung tissue has not yet been demonstrated. Earlier reports have linked CD8^+^CD28^-^ cells from peripheral blood and small airways samples of COPD patients with a decline in lung function (31) and increased steroid-resistance (7, 27, 32). These “effector-senescent” lymphocytes, often phenotypically CD8^+^CD45RA^+^CD28null, are characterized by impaired apoptosis and corticosteroid unresponsiveness (7, 16). *In vitro*, ciclosporin restored steroid sensitivity and reduced proinflammatory cytokine production in these cells, underscoring their pathogenic and potential therapeutic relevance.

Recently, Villaseñor-Altamirano et al. similarly reported enrichment of cytotoxic CD8^+^KLRG1^+^ TEMRA cells in mild-to-moderate COPD, whereas these cells were less abundant in severe disease (33). This discrepancy may reflect both disease stage and methodological differences: resection samples from early-stage COPD were profiled by single-cell RNA-seq, whereas severe COPD was represented by only two explanted lung samples analysed by bulk RNA-seq. Therefore, the results of our study add to these findings in terms of end-stage COPD, as CD8^+^ KLRG1^+^ TEMRA cells and CD8^+^CD28^-^CD27^-^ cells may represent overlapping populations on the spectrum of terminal differentiation, both contributing to cytotoxicity and tissue damage. In contrast, a recent study found an increased expression of CD28 and CD27 in hot spots in explanted COPD lungs (5). Further studies are needed to clarify the roles of CD8^+^CD28^-^CD27^-^ subsets and their potential as therapeutic targets.

In IPF, the potential role of CD8^+^CD28null T cells is intriguing. In our study, we observed a trend toward increased CD8^+^CD28^-^CD27^-^ cells compared with donor lungs. A prior report demonstrated a significant expansion of cytotoxic, senescent CD8^+^CD28null T cells in explanted IPF lungs, which promoted fibrosis in a murine model but were restrained by CTLA-4 and PD-1 signaling (16). These CD28null T cells were resistant to dexamethasone, potentially via downregulation of the glucocorticoid receptor and HDAC2, a mechanism also described in COPD (10, 29). More recently, single-cell RNA sequencing confirmed an increased abundance of CD8^+^ T cells in IPF lungs with enrichment of fibrosis-related pathways, further implicating this subset in disease pathogenesis (34).

We further observed a significant reduction of Tc17 (CCR6^+^CD8^+^) cells in COPD and IPF lung tissue compared with healthy controls. Prior studies reported increased Th17 cells in COPD, suggesting an imbalance between pro- and anti-inflammatory responses, although their precise role remains unclear (13–15, 35). Our data did not confirm a Th17 signal in COPD lung. In murine models, CCR6 deficiency or IL-23 blockade reduced cigarette smoke–induced emphysema, whereas anti-IL-23 treatment in severe asthma patients worsened outcomes, highlighting the complex role of the Th17 axis in airway disease (36, 37). In IPF, Th17 cells have been implicated in fibrosis, with attenuated disease in IL-17-deficient mice (15, 35). Tc17 cells have also been linked to emphysema progression in mice (8, 9). Consistent with our findings, explanted COPD lungs showed reduced CCL20 expression, a CCR6 ligand and chemoattractant for Th17/Tc17 cells, providing indirect support for the decreased Tc17 compartment observed in our study (38).

Finally, our results reveal two distinct alterations of DN T cell populations in COPD and IPF: the expansion of CD25^+^ DN subsets in COPD and the depletion of DN TRM cells across both diseases. To the best of our knowledge, these are novel observations in human lung tissue. iNKT DN subsets showed no differences between groups in our study. A threefold increase in CD25^+^ DN T cells in COPD is consistent with enhanced IL-2–pathway within the DN compartment; this increase could reflect either a compensatory regulatory response reported for some DN subsets or an activated effector pool contributing to disease (39). Conversely, reduced DN TRM (CD69^+^CD25^-^HLA-DR^-^) cells in both diseases may reflect impaired local immune surveillance, observed clinically, and/or stromal–antigenic dysregulation in chronic lung injury. Congruently, in a murine influenza model (40), NK1.1^-^ DN T cells localized in the lung parenchyma, exhibited a pre-activated TRM phenotype (CD44^+^CD69^+^CD103^+^), and expanded robustly after infection, underscoring the capacity of DN TRM to participate in acute, site-restricted immunity. More broadly, DN T cells have recently been linked to autoimmune and inflammatory diseases (22). Their role in immunoregulation and pathogenic processes remains controversial; however, under certain conditions they are proinflammatory, cytotoxic, and potentially corticosteroid-resistant. Given the paucity of prior lung-tissue data in COPD or IPF, our findings warrant further functional studies to define causality and therapeutic implications.

This study has several notable strengths. First, using healthy, transplantable lung tissue for comparison, rather than tissue from lung cancer resections, ensures that the underlying malignancy does not confound the findings. Additionally, all participants were nonsmokers at the time of lung explantation, which further minimizes the risk of confounding effects due to active smoking and smoking-induced inflammation. A significant advantage of this study is the ability to compare changes in lung tissue between advanced COPD patients and IPF patients within the same study protocol, allowing for the exploration of similarities and differences between these conditions. We also included a limited number of samples from IPAH lungs, where data on T cell subsets in lung tissue are scarce. However, our study has several limitations. Our control group included predominantly men due to clinical and logistical constraints in tissue availability, which is a limitation. However, the modest nature of known sex-related immune differences suggests minimal impact on our primary findings (41). Furthermore, cell viability was not assessed directly in experimental samples due to limited lymphocyte numbers in lung tissue. Instead, we evaluated viability in separately processed lung resections using identical protocols, which confirmed high post-processing cell viability. However, the absence of per-sample viability assessment remains a limitation. Similarly, we used a limited marker panel to preserve sufficient cell numbers for robust analysis. While we successfully identified specific cell subtypes within the pretransplant lung samples via flow cytometry of tissue T cells, the study did not investigate intracellular cytokine expression, T-cell receptor sequencing, or individual cellular expression profiles, highlighting the use of single-cell sequencing as a critical approach for future research.

## Conclusions

The current study, to the best of our knowledge, represents the largest flow cytometric assessment of COPD and IPF lung transplant samples, along with a control group of healthy lungs from donors. These findings expand our understanding of how the T-cell response and dysregulation may affect end-stage COPD and IPF in humans and raise new questions about the specific role of Th1 lymphocytes, terminally differentiated cytotoxic T cells, Tc17 lymphocytes and DN subsets in the progression and terminal similarities between these two diseases. The potential benefit of pinpointing T-cell pathogenetic mechanisms may lead to new targeted interventions and therapeutic improvements in COPD and IPF patients.

## Data Availability

The raw data supporting the conclusions of this article will be made available by the authors, without undue reservation.
