# Supplementary material for: Elevated Th1 and terminally differentiated cytotoxic T cells with suppressed Tc17 lymphocytes in lung tissue of advanced COPD and IPF patients undergoing lung transplantation
Source: Front Immunol. 2025 Oct 14;16:1646711. doi: 10.3389/fimmu.2025.1646711 (PMC12558995; doi:10.3389/fimmu.2025.1646711)
Supplement: Supplementary file 1 [file Supplementaryfile1.docx]

**Supplementary Material**

Title: Elevated Th1 and terminally differentiated cytotoxic T cells with suppressed Tc17 lymphocytes in lung tissue of advanced COPD and IPF patients undergoing lung transplantation

Authors: Irena Šarc^1,2^, Matthias Zimmermann^3,4^, Ana Koren^1^_,_ Luka Dejanović, Matija Rijavec ^1,5^_,_ Hendrik J Ankersmit^3,6^, and Peter Korošec^1,7^

^1^University Clinic of Respiratory and Allergic Diseases, Golnik, Slovenia,

^2^Medical Faculty, University of Ljubljana, Ljubljana, Slovenia,

^3^Christian Doppler Laboratory for Cardiac and Thoracic Diagnosis and Regeneration, Medical University of Vienna, Austria,

^4^Department of Oral and Maxillofacial Surgery, Medical University of Vienna, Vienna, Austria,

^5^Biotechnical Faculty, University of Ljubljana, Ljubljana, Slovenia,

^6^Division of Thoracic Surgery, Department of Surgery, Medical University of Vienna, Vienna, Austria,

^7^Faculty of Pharmacy, University of Ljubljana, Ljubljana, Slovenia

**Correspondence**: Irena Šarc, MD, University Clinic of Respiratory and Allergic Diseases Golnik; Golnik 36, 4204 Golnik, Slovenia; irena.sarc@klinika-golnik.si

**Supplementary methods**

**Cell viability testing**

Viability of the cells was tested on three samples. All samples were collected from clinically indicated lung resections. Lung tissue was processed at the University Clinic Golnik. All the samples were submerged in a transport medium consisting of ice-cold RPMI-1640 (Sigma‒Aldrich, St. Louis, MO) supplemented with 1% bovine serum albumin (BSA, Miltenyi Biotec, Germany), 1% L-glutamine, and 1% penicillin-streptomycin (both from Sigma‒Aldrich). The tissue was then finely minced into small pieces using a scalpel, washed, and enzymatically degraded with 20 µg/ml collagenase II (Gibco, USA) for 2 hours at 37°C with mixing every 15 minutes. The cell suspension was then filtered through a 70-μm nylon strainer (BD Falcon, USA).

Cells from Donor 1 were washed twice and finally resuspended in 500 µL PBS. PI viability dye (Thermo Fisher Scientific, USA) was then added to the suspension and incubated for 10 minutes at room temperature. Samples were acquired within 2 hours using a BF FACSCanto II flow cytometer.

Cells from Donors 2 and 3 were washed twice, resuspended in transport medium, and incubated overnight at 4 °C. Following overnight incubation cells were washed twice with PBS and finally resuspended in 500 µL PBS. PI viability dye (Thermo Fisher Scientific, USA) was added to the suspension and incubated for 10 minutes at room temperature. Samples were acquired within 2 hours using a BD FACSCanto II flow cytometer.

**Supplementary results**

**Cell viability**

Three samples were assessed for cell viability using the PI marker. Sample 1 (donor 1) was stained for viability fresh, without overnight incubation in transport media, unlike the other two samples (donors 2 and 3), which were incubated overnight in transport media prior to analysis. All samples demonstrated high cell viability, irrespective of the protocol: Sample 1 had 99.7% live cells, Sample 2 had 98.7%, and Sample 3 had 99.6% (**Figure S1**).

**Figure S1**: Cell viability assessment using propidium iodide (PI) staining.

Sample 1 (donor 1) was stained fresh, whereas Samples 2 and 3 (donors 2 and 3) were incubated overnight in transport medium, following the same protocol as the experimental patients samples, before staining.
